# Investigation of preclinical pharmacokinetics of *N*-demethylsinomenine, a potential novel analgesic candidate, using an UPLC-MS/MS quantification method

**DOI:** 10.3389/fchem.2023.1222560

**Published:** 2023-07-06

**Authors:** Lulu Yu, Xunjia Qian, Yiheng Feng, Yujian Yin, Xiao-Dan Zhang, Qianqian Wei, Liyun Wang, Weiwei Rong, Jie-Jia Li, Jun-Xu Li, Qing Zhu

**Affiliations:** ^1^ School of Pharmacy, Nantong University, Nantong, Jiangsu, China; ^2^ Provincial Key Laboratory of Inflammation and Molecular Drug Target, Nantong, Jiangsu, China; ^3^ Center for Neural Developmental and Degenerative Research of Nantong University, Institute for Translational Neuroscience, Affiliated Hospital 2 of Nantong University, Nantong, Jiangsu, China

**Keywords:** pharmacokinetics, bioavailability, N-demethylsinomenine, UPLC-MS/MS, rats

## Abstract

**N-** Demethylsinomenine (NDSM), the *in vivo* demethylated metabolite of sinomenine, has exhibited antinociceptive efficacy against various pain models and may become a novel drug candidate for pain management. However, no reported analytical method for quantification of **N-** Demethylsinomenine in a biological matrix is currently available, and the pharmacokinetic properties of **N-** Demethylsinomenine are unknown. In the present study, an ultra-high performance liquid chromatography with tandem mass spectrometry (UPLC-MS/MS) method for quantification of **N-** Demethylsinomenine in rat plasma was developed and utilized to examine the preclinical pharmacokinetic profiles of **N-** Demethylsinomenine. The liquid-liquid extraction using ethyl acetate as the extractant was selected to treat rat plasma samples. The mixture of 25% aqueous phase (0.35% acetic acid-10 mM ammonium acetate buffer) and 75% organic phase (acetonitrile) was chosen as the mobile phases flowing on a ZORBAX C18 column to perform the chromatographic separation. After a 6-min rapid elution, NDSM and its internal standard (IS), metronidazole, were separated successfully. The ion pairs of 316/239 and 172/128 were captured for detecting **N-** Demethylsinomenine and IS, respectively, using multiple reaction monitoring (MRM) under a positive electrospray ionization (ESI) mode in this mass spectrometry analysis. The standard curve met linear requirements within the concentration range from 3 to 1000 ng/mL, and the lower limit of quantification (LLOQ) was 3 ng/mL. The method was evaluated regarding precision, accuracy, recovery, matrix effect, and stability, and all the results met the criteria presented in the guidelines for validation of biological analysis method. Then the pharmacokinetic profiles of **N-** Demethylsinomenine in rat plasma were characterized using this validated UPLC-MS/MS method. **N-** Demethylsinomenine exhibited the feature of linear pharmacokinetics after intravenous (*i.v.*) or intragastric (*i.g.*) administration in rats. After *i. v.* bolus at three dosage levels (0.5, 1, and 2 mg/kg), **N-** Demethylsinomenine showed the profiles of rapid elimination with mean half-life (T_1/2Z_) of 1.55–1.73 h, and extensive tissue distribution with volume of distribution (V_Z_) of 5.62–8.07 L/kg. After *i. g.* administration at three dosage levels (10, 20, and 40 mg/kg), **N-** Demethylsinomenine showed the consistent peak time (T_max_) of 3 h and the mean absolute bioavailability of **N-** Demethylsinomenine was 30.46%. These pharmacokinetics findings will aid in future drug development decisions of **N-** Demethylsinomenine as a potential candidate for pain analgesia.

## 1 Introduction

Chronic pain usually refers to pain that persists or relapses for more than 3 months (Treede et al., 2019), which affects the physical and psychological health of patients in their daily life (Attal et al., 2018). Conventional oral analgesics are often chosen as the primary treatment for their fast action, low cost, and relative safety (Hylands-White et al., 2017). Although there are many analgesics available for clinical use, they all have some shortcomings such as limited efficacy or unexpected effects. Non-steroidal anti-inflammatory drugs (NSAIDs) are usually used as a starting medicine in treating pain, but they have limited efficacy against neuropathic pain and have a range of adverse effects related to the gastrointestinal, renal, and cardiovascular systems (Harirforoosh et al., 2013). Opioids are the most effective analgesics, but their broad unexpected effects can be serious and even lethal (Szigethy et al., 2018). Therefore, there is an urgent clinical need to discover new and effective analgesics with fewer side effects for the control of pain.

Traditional Chinese medicine (TCM) has a long history of human use and medicinal plants have been the sources of many successful modern medicines. Modern research on TCM has mostly focused on extracting and studying the pharmacology and mechanisms of ingredient compounds in TCM. Previous studies have found that sinomenine, a morphinan alkaloid, has significant analgesic properties in multiple chronic pain models (Zhao et al., 2012; Gao et al., 2013; Gao et al., 2014; Pertovaara, 2014; Zhu et al., 2016). However, clinical use of sinomenine is limited by its unexpected effects such as allergic and gastrointestinal reactions caused by strong histamine release and sedative effects (Zhang et al., 2018). *N*-Demethylsinomenine (NDSM), the demethylated derivative of sinomenine, is one of the bioactive ingredients of traditional Chinese herb *Sinomenium acutum*, and also an active metabolite of sinomenine (Cheng et al., 2007; Yi et al., 2012). Previous studies in our laboratory have shown that NDSM has analgesic effects against postoperative pain (Ou et al., 2018) and chronic pain (Zhou et al., 2021) without a sinomenine-like peripheral allergic reaction, and its sedative effect is far less than sinomenine (see Supplementary Figure S1 and Supplementary Table S1), suggesting that NDSM may be more advantageous than sinomenine for pain management.

Given the importance of pharmacokinetic profiling in preclinical drug development (Singh, 2006; Zheng et al., 2022), we aimed to continue our research into NDSM by extending the understanding of its pharmacokinetic profiles to rats. In the present study, a fast, accurate, and sensitive ultra-high performance liquid chromatography with tandem mass spectrometry (UPLC-MS/MS) method was established and certified to quantitatively detect NDSM in rat plasma. This method was utilized to investigate pharmacokinetics of NDSM after successful intravenous or oral administration.

## 2 Materials and methods

### 2.1 Chemicals and reagents

NDSM (purity >98.0% by HPLC purity analysis) was synthesized in-house and confirmed by NMR spectroscopy, which conformed to a previous report (Cheng et al., 2007). The internal standard (IS) metronidazole (purity >98.0%) was purchased from Beijing InnoChem Science and Technology Co., Ltd. (Beijing, China). Methanol and acetonitrile provided by Tedia (Fairfield, OH, United States) meet the requirements for mass spectrometry analysis. Chromatographic grade acetic acid, ammonium acetate, and ethyl acetate were purchased from Aldrich-Sigma (St Louis, MO, United States of America). Deionized water was provided by a Milli-Q ultrapure water preparation system (Millipore, MA, United States). Other reagents were all analytical grades.

### 2.2 Animals

Male and female Sprague-Dawley rats, weighing 200–250 g, were obtained from the Laboratory Animal Center of Nantong University (Nantong, China). Animals were acclimated under a controlled environment including humidity between 50%–70%, temperature of 25°C, and a 12-h light/dark cycle. All rats were subjected to overnight fasting with free access to water prior to the experiments. The animal protocols were approved by the Institution for Animal Care and Use Committee of Nantong University and followed the United States National Institutes of Health Guide for the Care and Use of Laboratory Animals (eighth edition).

### 2.3 Instruments and UPLC-MS/MS conditions

The UPLC-MS/MS system comprised an Agilent 1290 series UPLC system (Agilent Technologies, CA, United States) and a SCIEX 5500 QTrap triple quadrupole/linear ion trap hybrid mass spectrometer outfitted with an electrospray ionization (ESI) source (Applied Biosystems/MDS Sciex, ON, Canada). Equipment control, data, and analysis were acquired and manipulated by the Analyst 1.6.2 software (Applied Biosystems/MDS Sciex, ON, Canada).

Chromatography was conducted on an Agilent Eclipse XDB-C18 column (5 μm, 4.6 × 150 mm) attached to a guard column, Agilent Eclipse XDB-C18 (5 μm, 4.6 × 12.5 mm). The temperature of the chromatographic column was kept at 30°C, and a 6-min isocratic elution program was conducted under aqueous phase A (10 mM ammonium acetate containing 0.35% acetic acid) and organic phase B (acetonitrile): 0–6 min (75% B). The flow rate was set as 0.3 mL/min, and 2 µL of analyte was injected into the analysis system. Mass spectrometry under ESI positive ion mode was conducted under the settings optimized as follows: nitrogen as the nebulizer gas, gas ion source 1 (50 psi), gas ion source 2 (50 psi), source temperature (350°C), and ion spray voltage (4000 V). Collision energy (CE), declustering potential (DP), collision exit potential (CXP), and entrance potential (EP) were optimized at 27, 160, 12, and 10 V for NDSM and 19, 103, 9, and 10 V for IS, respectively.

Quantitative detection was conducted based on multiple reaction monitoring (MRM) mode with parent-daughter ion transitions of m/z 316→239 for NDSM and m/z 172→128 for IS, respectively.

### 2.4 Standard solution and quality control (QC) samples

Stock solutions of NDSM (1 mg/mL) and IS (1 mg/mL) were prepared accurately in methanol and stored at −20°C. The standard working solutions (0.03, 0.1, 0.3, 1, 3, and 10 μg/mL) and QC solutions (0.06, 0.6, and 8 μg/mL) were acquired by serially diluting the stock solution of NDSM in methanol. The plasma standard samples (3, 10, 30, 100, 300, and 1000 ng/mL) used for the calibration curve were prepared by mixing 90 μL of blank rat plasma and 10 μL of standard working solutions. QC samples (6, 60, and 800 ng/mL, respectively) were prepared in the same way. The IS working solution was prepared in methanol to yield an ultimate concentration of 30 ng/mL.

### 2.5 Sample preparation

Ethyl acetate was selected as the extractant for liquid-liquid extraction (LLE). First, 100 µL of plasma samples spiked with 10 µL of IS working solution and 10 µL of 1M NaOH were vortexed for 1 min to mix. Subsequently, 1000 µL of ethyl acetate were added in two batches, each swirled for 8 min, followed by being centrifuged at 10,000 rpm for 10 min. The supernatant was transferred into a new centrifuge tube and then dried under nitrogen stream with water bath at 30°C. The residue was redissolved with 110 µL of 75% acetonitrile/water and then was centrifuged (12,000 rpm for 12 min) again. Finally, 80 µL of the supernatant was moved into an autosampler vial for analysis.

### 2.6 Method validation

To demonstrate the rationality of the UPLC-MS/MS method, the sensitivity, specificity, linearity, precision and accuracy, matrix effects, extraction recovery, and stability of NDSM in rat plasma were validated following the regulations and standards outlined by the Food and Drug Administration (FDA) in the United States (Kadian et al., 2016).

#### 2.6.1 Specificity and sensitivity

The chromatograms of mixed blank plasma from six different healthy rats and six replicated standard plasma samples at the lower limit of quantification (LLOQ) spiked with IS (at final concentration of 30 ng/mL) was compared to assess the specificity and sensitivity of this method. In addition, the *in vivo* plasma samples after an oral dosage of NDSM (20 mg/kg) were also detected to assess whether this method with validated LLOQ was sensitive enough to cover all the *in vivo* plasma samples.

#### 2.6.2 Linearity

In order to evaluate the linearity, six NDSM concentrations (3–1000 ng/mL) were calibrated and detected for six uninterrupted days. The linear equation was built up using least squares linear regression by plotting the peak area ratios of NDSM to IS on the ordinate (*Y*-axis) *versus* the concentration of NDSM on the abscissa (*X*-axis). Plasma samples spiked with NDSM at a final concentration of 3 ng/mL (LLOQ) represented the sensitivity of this method with the signal/noise (S/N) ≥ 10. The detection limit of NDSM was determined as 1 ng/mL (S/N ≥ 3).

#### 2.6.3 Precision and accuracy

To evaluate the precision and accuracy of the UPLC-MS/MS method, simulated plasma samples were analyzed at LLOQ and QC levels (3, 6, 60, and 800 ng/mL) with five replicates in each level on the same day and on three consecutive days, respectively. For LLOQ level, the precision (expressed as percent form of relative standard deviation, %RSD) must be within ±20%, and the accuracy (expressed as percent form of relative error, %RE) must be within ±20%. For QC levels, the precision should be within 15%, and the accuracy should range between −15% and 15%.

#### 2.6.4 Recovery and matrix effect

The extraction recovery was tested by comparing the peak areas of NDSM from the extracted QC samples with those from the extracted blank plasma samples spiked with standard solutions at equivalent QC levels (6, 60, and 800 ng/mL). The matrix effect was investigated by comparing the peak areas from the extracted blank plasma samples spiked with standard solutions to those from methanol standard solutions at equivalent QC levels (6, 60, and 800 ng/mL). All QC samples were run in five replicates. The evaluation method for matrix effect and recovery of IS (30 ng/mL) was consistent with NDSM.

#### 2.6.5 Stability

The stability of NDSM in the biological matrix was analyzed at QC levels under various storage conditions: untreated samples preserved at room temperature for 6 h (short-term stability), or in the −20°C for 30 d (long-term stability), or after three freeze-thaw cycles from −20°C to 25°C (freeze-thaw stability), and processed samples in the 4°C autosampler for 24 h (autosampler stability). Five independent samples were prepared for each concentration, and the %RSD and %RE were calculated. The judgment that samples were stable could be made if the deviation between the measured concentration and the nominal concentration was within 15.0%.

#### 2.6.6 Dilution integrity

Simulated plasma samples were prepared at a concentration of 2000 ng/mL, which was higher than the upper limit of quantification (ULOQ). The simulated plasma samples were diluted with blank rat plasma. The precision and accuracy of the diluted samples were not affected.

### 2.7 Pharmacokinetic application

To evaluate the bioavailability and pharmacokinetic profiles of NDSM, the plasma concentration of NDSM was measured after a single oral or intravenous administration at different dose levels. Six groups of rats were allocated randomly, each consisting of six rats, three male and three female, by oral gavage (10, 20, 40 mg/kg) or intravenous injection (0.5, 1, 2 mg/kg), respectively. Blood samples (approximately 0.4 mL) were collected from the tail vein into anticoagulant tubes at 0.083, 0.167, 0.333, 0.75, 1, 2, 3, 4, 6, 8, 12, and 24 h after intragastric administration (*i.g.*), and at 0.033, 0.116, 0.25, 0.5, 0.75, 1, 1.5, 2, 4, 6, and 8 h after intravenous administration (*i.v.*). According to the results from the concentration-dependent time curves obtained from our preliminary experiment, we designed this blood collection schedule to include all the absorption, distribution, and elimination phases. During the blood collection, the internal blood volume of rats was maintained by intravenously injecting 1 mL of normal saline to rats every three sampling intervals. Blood samples were centrifuged at 4000 rpm for 10 min, and then plasma supernatants were stored at −20°C.

The pharmacokinetics parameters were calculated by the PKSolver software with a non-compartmental model (Zhang et al., 2010). The oral absolute bioavailability was calculated according to a previous report (Zheng et al., 2022). Statistical analyses were performed by one-way analysis of variance (ANOVA) followed by Tukey’s *post hoc* analysis using GraphPad Prism 8.4 Software (San Diego, CA).

## 3 Results and discussion

### 3.1 Method development and optimization

#### 3.1.1 Mass parameters and chromatography conditions

**FIGURE 1:**
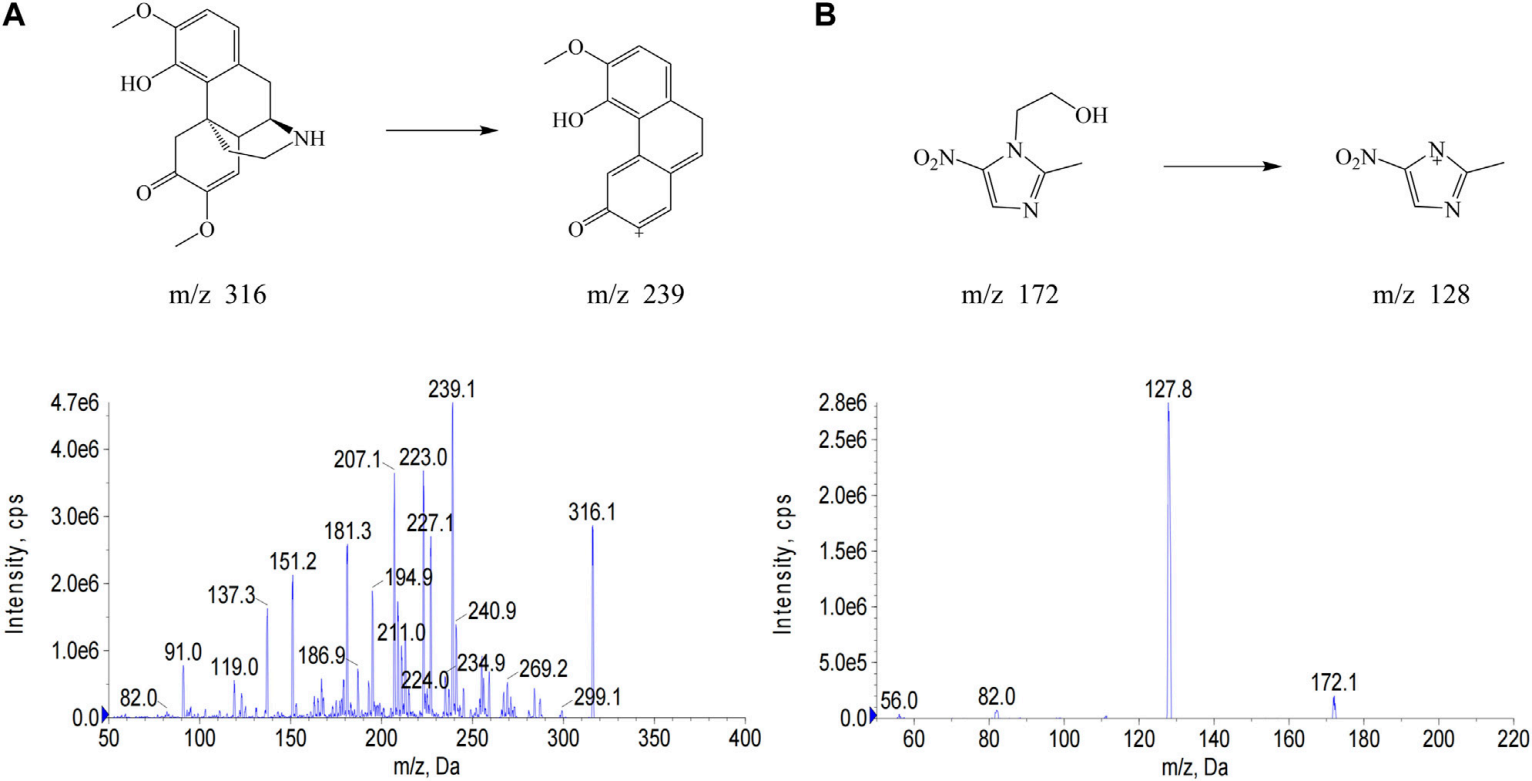
The chemical structure and full scan product ion mass spectra of *N*-demethylsinomenine (NDSM) **(A)** and the internal standard (IS) metronidazole **(B)** with monitoring at m/z 316→239 for NDSM and m/z 172→128 for IS.

In this study, metronidazole, which also has nitrogen-containing heterocycles, was selected as the IS to avoid the interference of NDSM metabolites. The quasi-molecular ion (Q1 scan mode) and the major product ion fragments (MS2 scan mode) of NDSM and IS were obtained by injecting their net standard solution in methanol into the mass spectrometer coupled with electrospray ion source (Figure 1). The MRM with the transitions at m/z of 316→239 and 172→128 were selected for quantification of NDSM and IS, respectively, owing to their good separation and better peak intensity. Better peak shape and suitable retention time were obtained while the mobile phase was 10 mM ammonium acetate containing 0.35% acetic acid and acetonitrile. The total running time was 6 min, which was relatively quick and could be suitable for the detection of a large number of samples.

#### 3.1.2 Comparison of the performance of sample preparation approaches

Endogenous compounds present in the biological matrix, such as phospholipids, proteins, and salts, can co-elute with the target analytes, and can interfere with mass spectrometric ionization, leading to ion enhancement or suppression (Desfontaine et al., 2018). We compared protein precipitation methods with methanol or acetonitrile (Wawrzyniak et al., 2018), salting-out assisted liquid-liquid extraction with ammonium acetate (Li et al., 2021), liquid-liquid extraction with ethyl acetate (Bao et al., 2017; Kanu, 2021), and solid-phase extraction (SPE) with C18 column (Lin et al., 2021).

After protein precipitation by adding three times the volume of methanol or twice the volume of acetonitrile to the simulated plasma sample containing IS, the extraction recovery rates of NDSM were higher than 95%, but both showed obvious suppressive matrix effect (4.68%), which led to poor sensitivity and so could not meet the requirements.

SPE is one of the key technologies for purifying complex biological fluids that can achieve good matrix effects (Lin et al., 2021). Acetonitrile containing 0.35% acetic acid was chosen as the eluent for C18 SPE column (Welch, Shanghai, China) to improve the elution capacity. Under a vacuum solid-phase extraction device (Merck, United States), we found that slowing down the elution rate and increasing the elution capacity of eluent could be beneficial for improvement of recovery, but it still could not achieve good extraction recovery (<60%).

Liquid-liquid extraction is a simple method with excellent extraction performance. In recent years, water-soluble solvent-assisted LLE methods, in which salts or sugars rather than hydrophobic solvents are used to treat biological samples, have been developed (Zhang et al., 2009). However, corrosion and clogging of mass spectrometry equipment caused by salt or sugar crystallization cannot be ignored. Compared to nonvolatile salts such as phosphate, this experiment used mass spectrometry-friendly ammonium acetate salting out assisted liquid-liquid extraction, but it was not effective in improving the recovery and matrix effect.

**FIGURE 2:**
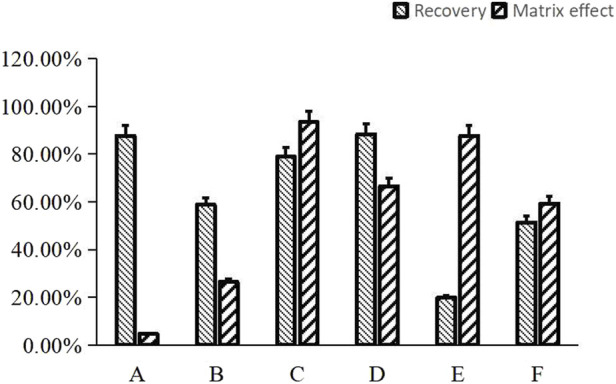
Comparison of the effect of different sample preparation approaches on matrix effect and recovery for NDSM. **(A)**, protein precipitation with acetonitrile showed high recovery rate (87.65% ± 19.10%), but matrix effect (4.68% ± 0.14%) did not meet the requirements; **(B)**, salting-out assisted liquid-liquid extraction with ammonium acetate showed poor recovery rate (58.75% ± 5.30%) and matrix effect (26.45% ± 3.07%); **(C)**, liquid-liquid extraction with ultimate concentration of 0.083M NaOH and ethyl acetate met the requirements for recovery rate (78.98% ± 2.74%) and matrix effect (93.43% ± 6.32%); **(D)**, liquid-liquid extraction with ultimate concentration of 0.154M NaOH and ethyl acetate exhibited high recovery rate (88.32% ± 5.42%), but the matrix effect (66.56% ± 1.88%) was not as good as method **(C)**; **(E)**, solid-phase extraction (SPE) of plasma sample anticoagulated by heparin exhibited low recovery rate (19.84% ± 1.65%); **(F)**, SPE of plasma sample anticoagulated by EDTA showed higher recovery rate (51.40% ± 14.80%) than method **(E)** but still did not meet the standard.

Previous studies have indicated that the addition of NaOH to plasma samples can slightly enhance the recovery of alkaloids (Xu et al., 2013). In this paper, 10 µL or 20 µL of 1 M NaOH was added into 100 µL plasma samples plus 10 µL IS solution to yield the ultimate concentration of 0.083 M or 0.154 M NaOH, then under these cases the recovery rates and matrix effects of NDSM were measured and compared. Finally, the results showed that twice liquid-liquid extraction using ethyl acetate to extract solvent when the plasma sample contained about 0.083 M NaOH can simultaneously achieve great recovery rate (78.98%) with no obvious matrix effect (93.43%), which met the requirements of detection (Figure 2).

### 3.2 Method validation

#### 3.2.1 Selectivity, specificity, and sensitivity

**FIGURE 3:**
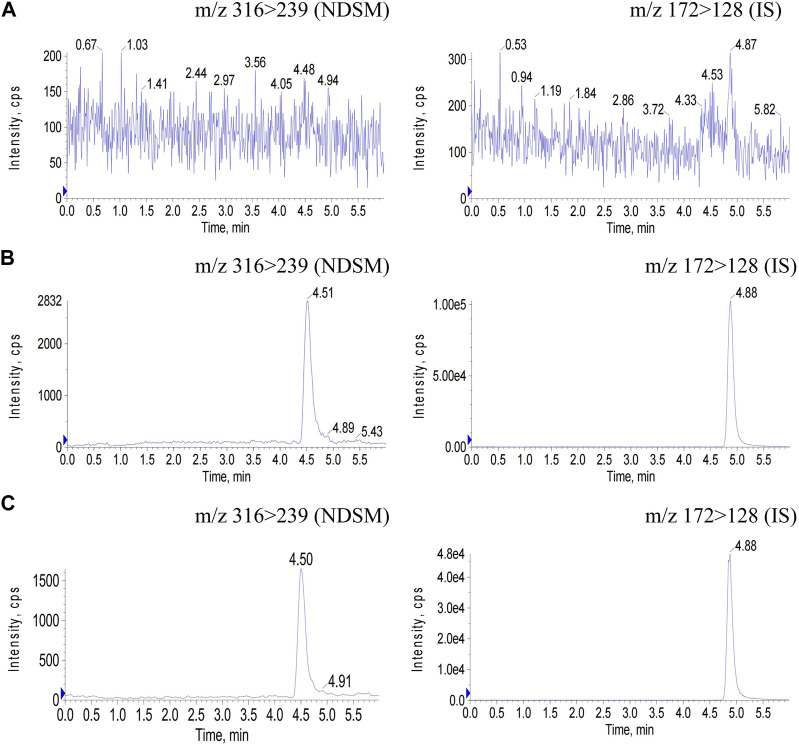
Chromatograms of NDSM (MRM 316/239) and IS (MRM 172/128) in rat plasma samples. **(A)** blank plasma sample; **(B)** blank plasma spiked with NDSM at the LLOQ level and IS; **(C)** plasma sample at 24 h after oral administration of NDSM (20 mg/kg). No interference peak in blank plasma samples was observed at the retention time of NDSM (4.5 min) and IS (4.8 min).

The chromatograms of blank plasma, blank plasma spiked with NDSM at the LLOQ level and IS, and rat plasma samples after oral administration of NDSM (20 mg/kg) were demonstrated in Figure 3. No endogenous interference in the plasma was observed at the retention time of NDSM and IS, and the peak area of the blank signal recorded at abovementioned retention times was less than 5% that of NDSM (LLOQ level) and IS (30 ng/mL), showing good selectivity and specificity. So, the LLOQ was validated at 3 ng/mL, which was sensitive enough for the detection of the last time-point sample concentration.

#### 3.2.2 Linearity and calibration curve

The standard curve exhibited superb linearity over the concentration range of 3–1000 ng/mL. The typical regression equation of the standard curve was Y = 0.00827X - 0.00652 (the correlation coefficient: 0.99955, weighting: 1/x).

#### 3.2.3 Precision and accuracy

Table 1 summarized the results of intra-day and inter-day precision and accuracy of NDSM at the LLOQ and QC sample levels. The precision was expressed by %RSD, and the accuracy was represented by %RE. Both the %RSD and %RE values were below 15%, suggesting the outcomes were satisfactory.

**TABLE 1:** Precision and accuracy of NDSM in rat plasma.

QC conc. (ng/mL)	Intra-day (n = 5)			Inter-day (n = 15, 3 days)		
	Mean ± SD ng/mL	Precision (RSD,%)	Accuracy (RE,%)	Mean ± SD ng/mL	Precision (RSD,%)	Accuracy (RE,%)
3 (LLOQ)	3.30 ± 0.33	10.05	9.93	3.25 ± 0.41	12.52	8.43
6.00	5.52 ± 0.42	7.68	−7.92	6.44 ± 0.71	10.96	7.31
60.00	62.43 ± 3.60	5.77	4.04	59.87 ± 4.20	7.01	−0.22
800.00	820.68 ± 29.51	3.60	2.58	787.65 ± 54.51	6.92	−1.54

#### 3.2.4 Recovery and matrix effect

The matrix effect and extraction recovery for three QC samples of NDSM and IS were shown in Table 2. The findings demonstrated that the mean extraction recovery rate ranged from 73.55% to 81.88% for NDSM and was 94.19% for IS, suggesting that a high rate of extraction recovery was achieved. Importantly, the extraction efficiency was independent of concentration and the precision was 2.41%–7.59% for NDSM and 2.38% for IS, suggesting it was stable and reproducible. Meanwhile, the mean matrix effect ranged from 92.95% to 103.96% for NDSM and was 89.73% for IS, which suggested no notable matrix effect on the determination.

**TABLE 2:** Extraction recovery and matrix effect of NDSM (n = 5).

	QC conc. (ng/mL)	Extraction recovery		Matrix effect	
		Mean ± SD (%)	RSD (%)	Mean ± SD (%)	RSD (%)
NDSM	6.00	73.55 ± 2.75	3.74	97.65 ± 10.73	10.99
	60.00	78.44 ± 1.89	2.41	92.95 ± 7.78	8.37
	800.00	81.88 ± 6.21	7.59	103.96 ± 2.78	2.68
IS	30.00	94.19 ± 2.24	2.38	89.73 ± 0.42	0.47

#### 3.2.5 Stability

The stability of short-term, long-term, freeze-thaw, and autosampler conditions was shown in Table 3. The accuracy (%RE) and precision (%RSD) of the stabilities were well within the acceptable limit (±15%), suggesting that there are no obvious problems in stability under the test conditions.

**TABLE 3:** Stability data of NDSM in rat plasma (n = 5).

Conditions	QC conc. (ng/mL)	Mean ± SD ng/mL	Precision (RSD,%)	Accuracy (RE,%)
Short-term stability (at room temperature for 6 h)	6.00	5.53 ± 0.43	7.80	−7.75
	60.00	57.39 ± 1.04	1.81	−4.35
	800.00	761.50 ± 43.19	5.67	−4.81
Autosampler stability (processed samples at 4 °C autosampler for 24 h)	6.00	6.57 ± 0.31	4.79	9.39
	60.00	63.62 ± 3.59	5.64	6.03
	800.00	821.64 ± 63.04	7.67	2.70
Long-term stability (at −20 °C for 30 days)	6.00	5.67 ± 0.72	12.74	−5.46
	60.00	57.37 ± 2.72	4.75	−4.38
	800.00	814.75 ± 33.02	4.05	1.84
Freeze-thaw stability (3 cycles)	6.00	5.74 ± 0.76	13.26	−4.34
	60.00	57.51 ± 1.66	2.88	−4.16
	800.00	793.01 ± 28.26	3.56	−0.87

#### 3.2.6 Dilution integrity and residue effect

The dilution integrity was measured when the plasma concentration of the *in vivo* sample was higher than the upper limit of quantification (ULOQ, 1000 ng/mL) of this method. The accuracy (%RE) and precision (%RSD) of the dilution integrity both were less than 15%, which met the requirements of quantitation. Furthermore, no obvious residue effect was observed when a blank sample was loaded after the calibration sample with the highest concentration.

### 3.3 Pharmacokinetics and bioavailability of NDSM

The UPLC-MS/MS method was developed and applied to study the pharmacokinetics of NDSM after intragastric administration (*i.g.*) and intravenous administration (*i.v.*) in rats. In our previous reports, intraperitoneal injection (*i.p.*) of 20 mg/kg NDSM exhibited significant antinociceptive effects against postoperative pain (Ou et al., 2018) and neuropathic pain (Zhou et al., 2021). In this study, three dosage levels of 10, 20, and 40 mg/kg were selected for *i. g.* administration*.* In a preliminary study, we found that the plasma concentration of many *in vivo* samples exceeded ULOQ (1000 ng/mL) if intravenous dosage was higher than 2 mg/kg. Thus, we chose three dosage levels at 0.5, 1, and 2 mg/kg for intravenous pharmacokinetic study.

**FIGURE 4:**
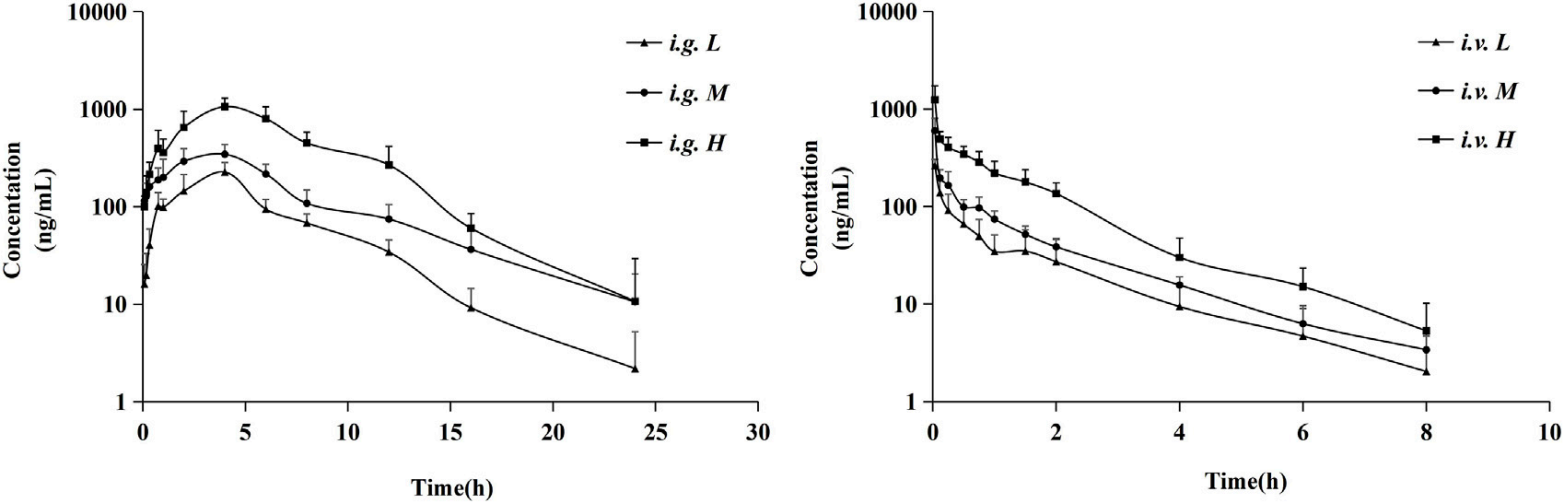
Mean plasma concentration profiles of NDSM after oral administration of 10 mg/kg (*i.g.* L), 20 mg/kg (*i.g.* M), and 40 mg/kg (*i.g.* H) and intravenous administration of 0.5 mg/kg (*i.v.* L), 1 mg/kg (*i.v.* M), and 2 mg/kg (*i.v.* H) (mean ± SD, n = 6). More details can be obtained in Table 4.

Figure 4 presented the average plasma concentration-time profiles and Table 4 summarized the main pharmacokinetic parameters of the non-compartment model for NDSM. In these concentration-time curves, the ratio of AUC_0-t_ to AUC_0-∞_ was greater than 90% and the concentration of the last time point sample was under 1/10 of C_max_, denoting that sampling schedule was appropriate and AUC_0-∞_ was reliable.

**TABLE 4:** The estimated mean pharmacokinetic parameters of NDSM after oral or intravenous administration (mean ± SD, n = 6).

Parameters (unit)	Oral administration			Intravenous administration		
	10 mg/kg	20 mg/kg	40 mg/kg	0.5 mg/kg	1 mg/kg	2 mg/kg
C_max_ (ng/mL)	226.42 ± 56.77	345.30 ± 89.68	1063.07 ± 242.20	-	-	-
C_2 min_ (ng/mL)	-	-	-	262.33 ± 42.62	598.98 ± 201.02	1243.18 ± 483.02
T_max_ (h)	3 ± 0	3 ± 0	3 ± 0	-	-	-
T_1/2Z_ (h)	3.45 ± 2.59	4.30 ± 1.12	2.92 ± 1.12	1.73 ± 0.71	1.70 ± 0.38	1.55 ± 0.76
AUC_(0-t)_ (ng/mL*h)	923.80 ± 163.96	1958.50 ± 517.98	5416.40 ± 1100.90	176.03 ± 82.48	301.90 ± 54.87	828.34 ± 209.16
AUC_(0-∞)_ (ng/mL*h)	954.70 ± 163.98	2033.39 ± 573.54	5583.96 ± 1203.10	185.52 ± 85.74	310.99 ± 56.69	843.11 ± 215.07
AUC_(0-t)_/AUC_(0-∞)_	0.97 ± 0.02	0.97 ± 0.03	0.97 ± 0.02	0.94 ± 0.06	0.97 ± 0.02	0.98 ± 0.02
MRT (h)	5.21 ± 1.61	6.30 ± 1.48	5.35 ± 1.35	1.81 ± 0.67	1.71 ± 0.38	1.52 ± 0.36
V_Z_ (L/kg)	-	-	-	7.99 ± 5.78	8.07 ± 1.98	5.62 ± 3.63
CL_Z_ (L/h/kg)	-	-	-	3.26 ± 1.51	3.34 ± 0.83	2.51 ± 0.67
F (%)	26.24% ± 4.91%	32.44% ± 6.73%	32.69% ± 7.15%	-	-	-

**FIGURE 5:**
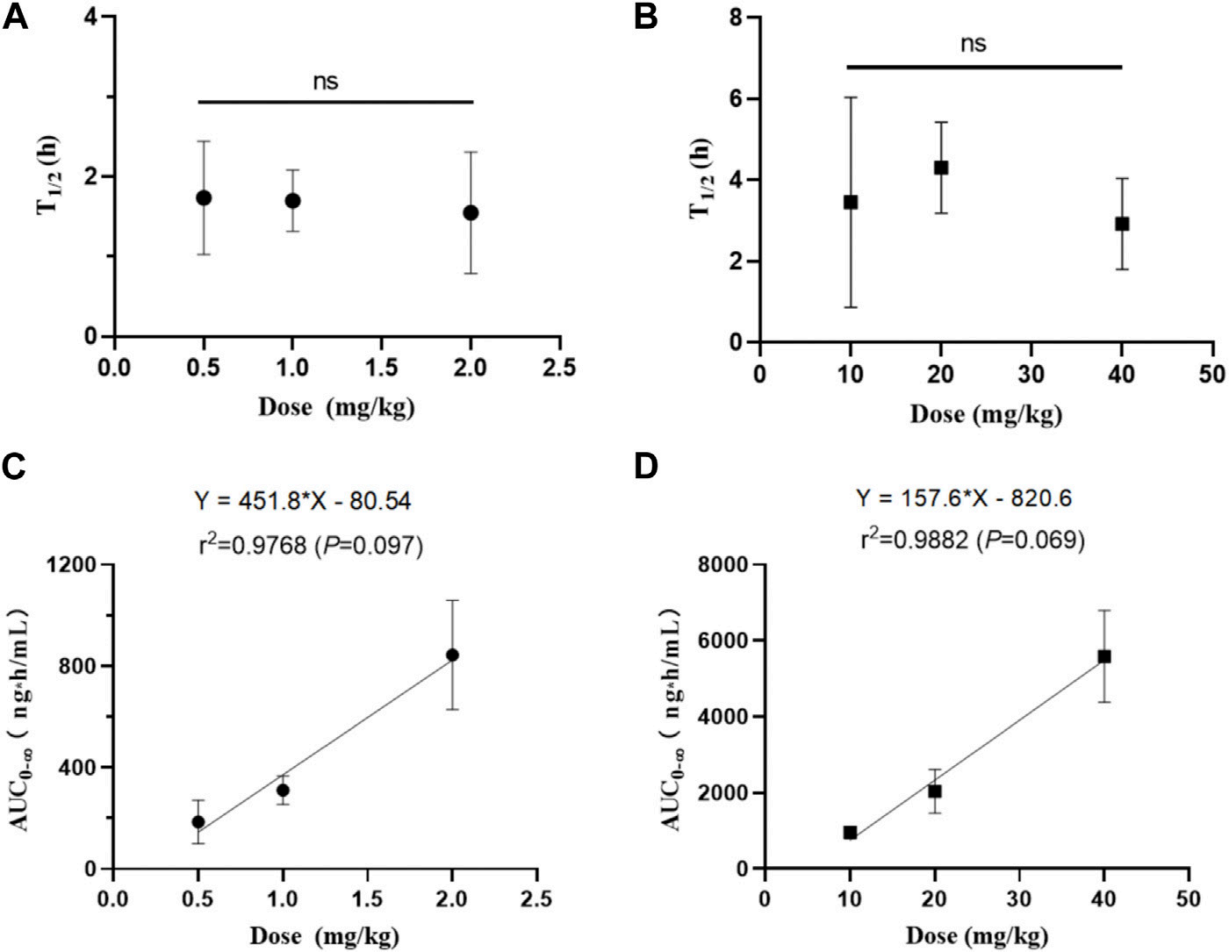
The relationship between half-life (T_1/2z_) and dose of NDSM after single intravenous **(A)** and oral administration **(B)**, and the relationship between the area under the plasma concentration-time curve (AUC) and dose of NDSM after single intravenous **(C)** and oral administration **(D)** (mean ± SD, n = 6). The word “ns” indicated no differences for the half-life among the three dose levels (*p >* 0.05). The regression equation of AUC from zero to infinite (AUC_0-∞_) *versus* dose was Y = 451.8X - 80.54 (*r* ^2^ = 0.9768, *p* = 0.0973) for intravenous administration, and Y = 157.6X - 820.6 (*r* ^2^ = 0.9768, *p* = 0.0693) for oral administration. These results indicated that the half-life of NDSM was independent of the dosage and did not extend with the increase of the dosage, while AUC_0-∞_ increased in proportion to the dose.

The pharmacokinetic profiles after *i. v.* administration at dosage of 0.5, 1, and 2 mg/kg showed similar average elimination half-life (T_1/2Z_ of 1.55–1.73 h), mean residence time (MRT of 1.52–1.81 h), volume of distribution (V_Z_ of 5.62–8.07 L/kg), and systemic clearance (CL_Z_ of 2.51–3.34 L/kg/h), which had no significant differences between different dosage levels (*p* > 0.05). Similarly, the average elimination half-life (T_1/2Z_ of 2.92–4.23 h) and mean residence time (MRT of 5.21–6.30 h) of the pharmacokinetic curves after oral administration of 10, 20, and 40 mg/kg also had no significant differences between different dosage levels (*p >* 0.05). We also analyzed the relationship of AUC from zero time to infinite (AUC_0-∞_) *versus* dose after intravenous and oral administration by linear regression analysis using GraphPad Prism 8.4 Software. As shown in Figure 5, results showed that the regression equation of AUC *versus* dose for intravenous administration was Y = 451.8X - 80.54 (*r* ^2^ = 0.9768, *p* = 0.0973), as well as Y = 157.6X - 820.6 (*r* ^2^ = 0.9768, *p* = 0.0693) for oral administration. According to the correlation coefficients (*r* ^2^) > 0.95 and the associated values of *p* > 0.05, it indicated a linear relationship between AUC and dose (Srinivas NR., 2015). These results indicated that the half-life of NDSM was independent of the dosage and did not extend with the increase of the dosage, while AUC_0-∞_ increased in proportion to the dose, suggesting that the elimination of NDSM was rapid and consistent with the linear kinetic characteristics.

The volume of distribution (V_Z_ of 5.62–8.07 L/kg) far exceeding the total body fluid volume (0.6 L/kg) of rats (Zheng et al., 2022) demonstrated that the tissue distribution and extravascular uptake of NDSM was extensive. The total clearance (CL_Z_ of 2.51–3.34 L/kg/h) of NDSM was close to the rat hepatic flow velocity (3.3 L/kg/h) (Zheng et al., 2022), suggesting a quick clearance rate in rats.

The oral absolute bioavailability of 10, 20, and 40 mg/kg NDSM was calculated as 26.24% ± 4.91%, 32.44% ± 6.73%, and 32.69% ± 7.15%, respectively. The mean oral absolute bioavailability of NDSM was 30.46%, suggesting its suboptimal oral absorption. Additionally, NDSM showed a consistent time for peak plasma concentration (T_max_) of 3 h after oral administration at the abovementioned three dosage levels. The oral half-life of NDSM (2.92–4.23 h) is longer than that of intravenous injection (1.55–1.73 h), which may be tentatively explained by the deconjugation of NDSM glucuronide through the enterohepatic circulation (Ohtani et al., 1994). In a previous study, the T_max_, T_1/2_ and oral bioavailability of rats after a single oral administration of 90 mg/kg sinomenine were 0.66h, 5.5h, and 79.6%, respectively, which demonstrated that sinomenine has fast oral absorption and high oral bioavailability (Liu et al., 2005a). Based on the longer half-life, a single oral administration of sinomenine (150 mg/kg) can maintain the effective drug concentration in rat plasma for a long time, but many adverse reactions were observed around 60 min after drug administration (Liu et al., 2005b). The studies on the metabolism and excretion of sinomenine found that sinomenine experienced phase I biotransformation and active hepatobiliary excretion, which was mainly regulated by P-glycoprotein (Tsai and Wu, 2003). NDSM, a phase I metabolite of sinomenine in urine, had a longer T_max_ (3 h) and a shorter T_1/2Z_ (2.92–4.23 h) than sinomenine after oral administration. Furthermore, the oral bioavailability of NDSM was less than half that of sinomenine. These changes may be due to the fact that the polarity and hydrophilicity was enhanced when sinomenine was metabolized to be NDSM. It was reported that sinomenine could not be transformed to demethylated metabolites by intestinal microbes *in vitro* due to its benzazepine structure (He et al., 2017). Whether the transmembrane absorption of NDSM in the stomach is less than that of sinomenine needs further investigation.

In addition, no gender differences were found in rats after intravenous or oral administration of NDSM (see Supplementary Tables S2–S7).

## 4 Conclusion

In this study, for the first time we have reported a rapid, simple, and sensitive UPLC-MS/MS method for the quantification of *N*-demethylsinomenine (NDSM) in rat plasma. Metronidazole was selected as IS to avoid confusion with potential metabolites and analogues of NDSM, improving the confidence of the assay. The method has a good linear relationship in the concentration range of 3–1000 ng/mL, while the LLOQ is 3 ng/mL. The method also meets the requirements in precision, accuracy, selectivity, and stability. The recovery rate and matrix effect can be satisfied by LLE with ethyl acetate twice. The method has been successfully applied to the preclinical pharmacokinetic study of NDSM in rats. By comparing AUC data from oral and intravenous administration, the mean oral absolute bioavailability of NDSM is determined as 30.46%. The current results provide useful data for further development of NDSM as a potential clinical candidate for the management of chronic pain.

## Data Availability

The raw data supporting the conclusion of this article will be made available by the authors, without undue reservation.
