# Supplementary material for: Investigation of preclinical pharmacokinetics of N-demethylsinomenine, a potential novel analgesic candidate, using an UPLC-MS/MS quantification method
Source: Front Chem. 2023 Jul 6;11:1222560. doi: 10.3389/fchem.2023.1222560 (PMC10359479; doi:10.3389/fchem.2023.1222560)
Supplement: Supplementary file 1 [file DataSheet1.PDF]

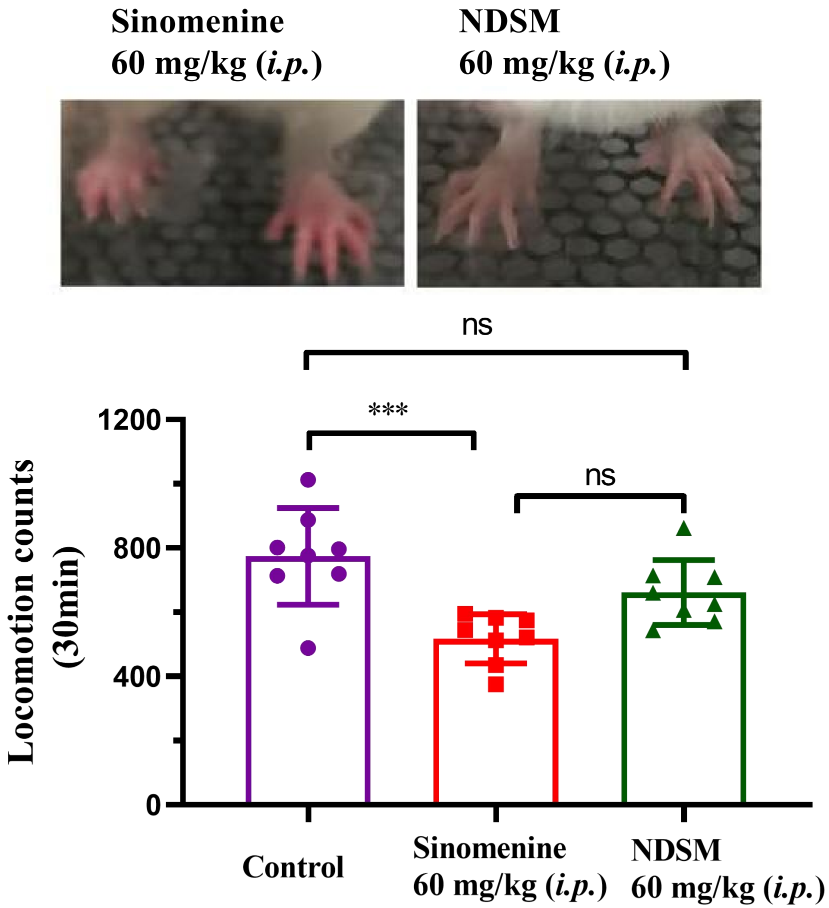


Table S1. Comparison of the effect of sinomenine and NDSM on the locomotor activities in rats

| Group | Control | Sinomenine 60 mg/kg (*i.p.*) | NDSM 60 mg/kg (*i.p.*) |
|---|---|---|---|
| Locomotion counts （30min） | 774.0±150.8 | 517.3±76.7 | 661.4±101.2 |

Figure S1. Comparison of the allergic reaction and sedative side effect of sinomenine versus *N*-demethylsinomenine (NDSM). Sinomenine showed obvious allergic reaction such as foot edema and produced sedative effect after intraperitoneal injection (*i.p.*) at dose of 60 mg/kg, but no obvious allergic reaction and sedation was observed after *i.p.*NDSM at the same dose. Data were expressed as mean±SD. * indicates a significant statistical difference form the Control group (***$P$ <0.001) ; ns indicates no significant difference between the NDSM group with the Control group or the Sinomenine group (*P*>0.05) (n=8). More details were provided in the Table S1.

Table S2-S7: No sex differences were found in pharmacokinetic parameters of *N*-demethylsinomenine between male and female rats.

S2: *i.v.* 0.5 mg/kg

| | male rats (n=3) | female rats (n=3) |
|---|---|---|
| $AUC_{(0-t)}$ (μg/L*h) | 247.52±32.95 | 104.53±24.24 |
| $AUC_{(0-\infty)}$ (μg/L*h) | 259.31±40.57 | 111.73±19.95 |
| MRT (h) | 2.15±0.70 | 1.47±0.54 |
| $T_{1/2Z}$ (h) | 1.83±0.46 | 1.63±1.01 |
| $V_Z$ (L/kg) | 5.06±0.79 | 10.92±7.55 |
| $CL_Z$ (L/h/kg) | 1.96±0.28 | 4.56±0.74 |
| $C_{2min}$ (μg/L) | 259.77±43.85 | 264.89±50.98 |

S3: *i.v.* 1 mg/kg

| | male rats (n=3) | female rats (n=3) |
|---|---|---|
| $AUC_{(0-t)}$ (μg/L*h) | 292.71±83.43 | 311.10±17.67 |
| $AUC_{(0-\infty)}$ (μg/L*h) | 296.63±83.72 | 325.34±20.16 |
| MRT (h) | 1.39±0.20 | 2.04±0.06 |
| $T_{1/2Z}$ (h) | 1.40±0.19 | 2.00±0.24 |
| $V_Z$ (L/kg) | 7.29±2.78 | 8.85±0.53 |
| $CL_Z$ (L/h/kg) | 3.60±1.21 | 3.08±0.18 |
| $C_{2min}$ (μg/L) | 657.86±227.43 | 540.11±197.22 |

S4: *i.v.* 2 mg/kg

| | male rats (n=3) | female rats (n=3) |
|---|---|---|
| $AUC_{(0-t)}$ (μg/L*h) | 752.02±164.87 | 904.66±254.39 |
| $AUC_{(0-\infty)}$ (μg/L*h) | 754.05±165.48 | 932.16±253.89 |
| MRT (h) | 1.23±0.04 | 1.81±0.26 |
| $T_{1/2Z}$ (h) | 0.96±0.16 | 2.13±0.63 |
| $V_Z$ (L/kg) | 3.77±0.77 | 7.46±4.70 |
| $CL_Z$ (L/h/kg) | 2.75±0.64 | 2.28±0.74 |
| $C_{2min}$ (μg/L) | 1309.46±526.19 | 1176.90±541.50 |

S5: *i.g.* 10 mg/kg

| | male rats (n=3) | female rats (n=3) |
|---|---|---|
| $AUC_{(0-t)}$ (μg/L*h) | 1030.95±213.58 | 852.36±108.72 |
| $AUC_{(0-\infty)}$ (μg/L*h) | 1052.32±207.96 | 889.61±127.56 |
| MRT (h) | 4.89±1.26 | 5.43±2.06 |
| $T_{1/2Z}$ (h) | 2.74±1.55 | 3.92±3.37 |
| $V_Z/F$ (L/kg) | 40.39±29.22 | 60.83±45.68 |
| $CL_Z/F$ (L/h/kg) | 9.69±1.92 | 11.41±1.75 |
| $C_{max}$ (μg/L) | 278.43±21.19 | 191.74±41.37 |

S6: *i.g.* 20 mg/kg

| | male rats (n=3) | female rats (n=3) |
|---|---|---|
| $AUC_{(0-t)}$ (μg/L*h) | 1790.47±176.95 | 2126.53±744.81 |
| $AUC_{(0-\infty)}$ (μg/L*h) | 1809.48±177.91 | 2257.31±800.19 |
| MRT (h) | 5.27±1.00 | 7.33±1.12 |
| $T_{1/2Z}$ (h) | 3.43±0.80 | 5.18±0.45 |
| $V_Z/F$ (L/kg) | 55.48±15.88 | 71.68±24.10 |
| $CL_Z/F$ (L/h/kg) | 11.13±1.11 | 9.52±2.81 |
| $C_{max}$ (μg/L) | 336.01±78.53 | 354.58±116.98 |

S7: *i.g.* 40 mg/kg

| | male rats (n=3) | female rats (n=3) |
|---|---|---|
| $AUC_{(0-t)}$ (μg/L*h) | 4669.16±639.32 | 6163.64±972.70 |
| $AUC_{(0-\infty)}$ (μg/L*h) | 4764.99±748.49 | 6402.93±1040.40 |
| MRT (h) | 4.75±1.13 | 5.95±1.48 |
| $T_{1/2Z}$ (h) | 2.31±0.47 | 3.52±1.35 |
| $V_Z/F$ (L/kg) | 28.22±6.07 | 33.10±15.51 |
| $CL_Z/F$ (L/h/kg) | 8.52±1.23 | 6.35±0.95 |
| $C_{max}$ (μg/L) | 1032.96±203.85 | 1093.17±319.97 |
